# Crystalline/Amorphous Blend Identification from Cobalt Adsorption by Layered Double Hydroxides

**DOI:** 10.3390/ma11091706

**Published:** 2018-09-13

**Authors:** Lin Chi, Zheng Wang, Yuan Sun, Shuang Lu, Yan Yao

**Affiliations:** 1 School of Civil Engineering, Harbin Institute of Technology, Harbin 150001, China; chilin8958@163.com (L.C.); wangz@163.com (Z.W.); qwertyburning@163.com (Y.S.); 2 Key Lab of Structures Dynamic Behavior and Control of the Ministry of Education, Harbin Institute of Technology, Harbin 150090, China; 3 Key Lab of Smart Prevention and Mitigation of Civil Engineering Disasters of the Ministry of Industry and Information Technology, Harbin Institute of Technology, Harbin 150090, China; 4 China Building Materials Academy, Beijing 100024, China; yy@cnbm.com.cn

**Keywords:** CaAl-Cl-LDH, cobalt, amorphous Al(OH)_3_

## Abstract

In this study, the adsorption behavior of CaAl-Cl layered double hydroxide (CaAl-Cl-LDH) with a controlled pH value (pH = 6) on Co(II) ions ([Co] = 8 mM) is investigated. The comprehensively accepted mechanism of cobalt adsorption on LDH is considered to be co-precipitation, and the final adsorbed products are normally crystalline Co-LDH. One unanticipated finding is that crystalline/amorphous blends are found in the X-ray diffraction (XRD) pattern of Co-adsorbed LDH. To shed light on the adsorption products and the mechanisms in the adsorption process of Co(II) in an aqueous solution by CaAl-Cl-LDH, a series of testing methods including Fourier-transform infrared spectroscopy (FT-IR), Scanning electron microscope (SEM), High-resolution transmission electron microscopy (HR-TEM), X-ray photoelectron spectroscopy (XPS), and inductively coupled plasma (ICP) are applied to clarify the interaction between cobalt and CaAl-Cl-LDH. According to the comprehensive analysis, the formation of the crystalline/amorphous blends corresponds to two adsorption mechanisms. The crystalline phases are identified as Co_6_Al_2_CO_3_(OH)_16_·4H_2_O, which is attributed to the co-precipitation process occurring in the interaction between Co(II) and CaAl-Cl-LDH. The formation of the amorphous phases is due to surface complexation on amorphous Al(OH)_3_ hydrolyzed from CaAl-Cl-LDH.

## 1. Introduction

Cobalt is widely used to manufacture rechargeable lithium ion batteries for mobile electronic devices, such as smartphones and laptops. Cobalt contamination problems are widely observed in various industries today [1]. Considering the usage of metallic cobalt and related salts in diverse industrial sectors, a higher concentration of cobalt may induce both environmental degradation and health problems [2]. Considering the high toxicity for human health, the cobalt concentration in wastewater is restricted to less than 1 mg/L by the emission standard of pollutants for the cobalt industry GB25467-2010. 

Layered double hydroxides (LDHs) are hydrotalcite-like clays, which are regarded as potential candidates for cationic absorbents in wastewater treatment [3,4]. LDH adsorbents play an adsorption role with respect to the emission of heavy metals present in different pollutants. Moreover, various LDH adsorbents have a specific and selective tendency in the adsorption of different heavy metals, including divalent cations (Cu^2+^, Ni^2+^, Zn^2+^, Cr^2+^, Ar^2+^, Cd^2+^, etc.), trivalent cations (Al^3+^, Fe^2+^, Cr^3+^, etc.), and anions (CO_3_^2−^, SO_4_^2−^, NO_3_^−^, Cl^−^, OH^−^, etc.) [3,5,6,7,8,9,10]. Generally, CaAl-Cl-LDHs can effectively absorb “target ions” from an aqueous medium in the following ways: co-precipitation, isomorphic substitution, and surface complexation and chelation [11]. For example, Co(II)-EDTA complexes can intercalate into Mg-Al LDH and go through a co-precipitation process [12]. Among multitudinous LDHs materials, Co-Al LDH can be applied as an electrode material for supercapacitors due to its excellent capacitance [13]. As Diao et al. states, MnO_2_/CoAl LDH nanocomposites exhibit both a relatively high specific capacity and a long cycling life [14]. According to the research of Han et al., core/shell nanoplatelet arrays composed by CoAl LDH-poly(3,4-ethylenedioxythiophene) (PEDOT) exhibit excellent energy storage capability [15]. 

There are relatively few studies devoted to studying cobalt removal by LDH, especially CaAl-Cl-LDH. The comprehensively accepted mechanism of cobalt adsorption on LDH is considered to be co-precipitation, and the final adsorbed products are normally crystalline Co-LDH [11]. One unanticipated finding is that crystalline/amorphous blends are noticed in the X-ray diffraction (XRD) pattern of Co-adsorbed LDH. To clarify the adsorption behaviors and the mechanisms in the adsorption process of Co(II) in an aqueous solution by CaAl-Cl-LDH, a series of testing methods including XRD, FT-IR, SEM, TEM, and X-ray photoelectron spectroscopy (XPS) are applied to clarify the interaction between cobalt and LDH. 

## 2. Materials and Methods

Analytical reagents and deionized water are applied during the solution preparation. CaAl-Cl-LDH is synthesized according to the precipitation method [7]. The main procedure of synthesizing CaAl-Cl-LDH begins with 0.25 M of NaAlO_2_ added to 0.5 M CaCl_2_ at a speed of 5 mL/min, and the mixture is heated at 50 °C, stirred at 300 rpm, and allowed to react for 1 h. The obtained precipitate is vacuum-filtrated and washed by deionized water until the soluble impurities can be removed entirely. Then, fine precipitate particles (45 μm) are obtained by milling after being oven-dried (Vacutherm, Thermo Fisher Scientific Inc., Carlsbad, CA, USA) at 50 °C for 24 h. 

A total of 0.2 g CaAl-Cl-LDH is added into 50 mL specific Co(NO_3_)_2_ ([Co] = 8 mM, pH = 6.0) solution, followed by a shaking procedure (150 rpm) in a shaking water bath at 25 ± 1 °C. At the selected time intervals (0.25, 0.5, 0.75, 1, 1.5, 2, 4, 8, and 24 h), 3 mL of supernatant is filtered through a 0.22-μm membrane filter for the analysis of Ca(II) and Co(II) concentrations. The Ca(II) and Co(II) concentrations are determined using inductively coupled plasma-mass spectrometry equipment (XSeries II ICP-MS, Thermo Fisher Scientific Inc., Carlsbad, CA, USA). All concentration results from the ICP-MS test are the average of two test runs. The pH is monitored by using a pH meter (Sartorius PB-10, Sartorius Inc., Goettingen, Germany).

The crystal structure of synthesized CaAl-Cl-LDH before and after Co(II) adsorption at a controlled pH value (pH = 6) are characterized by X-ray diffraction (XRD, X’Pert PRO MPD, Malvern Panalytical Inc., Malvern, UK) with CuK_α_ radiation in the 2θ range of 10–90° at the scanning rate of 0.02 °/s. The morphology and crystalline observations of the LDHs are undertaken by field scanning electron microscopy (FE-SEM, Hitachi S-4800, Hitachi Inc., Tokyo, Japan) with an energy dispersive X-ray spectroscope (EDS, Quantax75, Bruker Optik GmbH Inc., Ettlingen, Germany) attached, and high-resolution transmission electron microscopy (HR-TEM, Tecnai G2 F30, FEI Inc., Hillsboro, OR, USA) operated at 200 kV. The surface electronic properties of the CaAl-Cl-LDH are measured by X-ray photoelectron spectroscopy (XPS, ESCALAB 250Xi, Thermo Fisher Scientific Inc., Carlsbad, CA, USA). N_2_ adsorption–desorption isotherms are examined using a MicromeriticsASAP-2020 Instrument (MicromeriticsASAP-2020, Micromeritics Instrument Inc., Norcross, GA, USA) at 77 K. The LDHs are further studied by Fourier transform infrared spectroscopy (FT-IR, Nicolet iS50, Thermo Fisher Scientific Inc., Carlsbad, CA, USA) using KBr pellets ranging from 400–4000 cm^−1^.

## 3. Results

**Figure 1:**
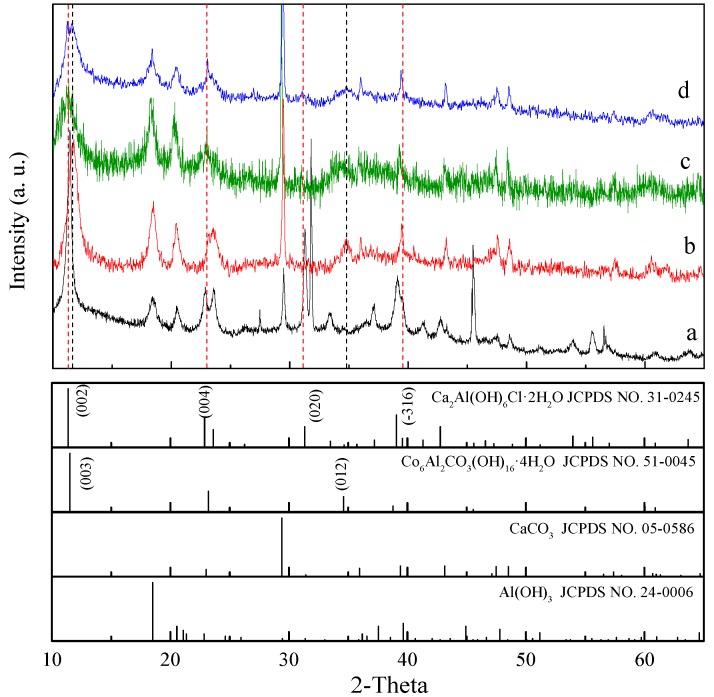
X-ray diffraction (XRD) patterns of original layered double hydroxides (LDHs) and Co-adsorbed LDHs at different pH values. (a) CaAl-Cl-LDHs; (b) Co-LDH at pH = 3; (c) Co-LDH at pH = 6; (d) Co-LDH at pH = 12.

The XRD patterns of CaAl-Cl-LDH before and after Co adsorption at different pH values are illustrated in Figure 1. As shown in Figure 1a, the observed diffraction peak positions are all in good agreement with those of CaAl-Cl-LDH (JCPDS No 31-0245), with a stoichiometric formula of Ca_4_Al_2_(OH)_12_Cl_2_·4H_2_O. Furthermore, additional peaks for CaCO_3_ polymorphs are observed, which is due to the carbonation of insufficiently consumed Ca ions. In Figure 1, bumps which indicate the presence of amorphous phases, most likely amorphous Al(OH)_3_, are also visible. This is partially due to the abundant introduction of Al ions during the synthesis process of CaAl-Cl-LDH. 

Generally, the typical variation in the basal spacing distances of the layered double hydroxide structure is usually proven to be due to some kind of reconstruction within the CaAl-Cl-LDHs during the divalent cations adsorption process [5]. As shown in Figure 1b, for the Co-adsorbed LDHs (Co-LDH) under the condition of acidity (pH = 3), the main diffraction peaks of the Co-LDH slightly shift towards 11.53°. The diffraction peaks at 11.67° and 46.76° of the Co-LDH corresponding to the 003 and 012 planes can be well-indexed to CoAl-Cl-LDH, according to the literature [13]. This is mainly due to the substitution of Co^2+^ by soluble cations in the acidic media. As for the XRD pattern of the synthesized LDH in alkali water solutions (pH = 12) in Figure 1d, the basal spacing parameters d_003_ and d_012_ of Co-LDH in alkaline media are 7.67° and 2.59°, respectively. In alkaline media, the introduced CO_3_^2−^ is inclined to replace Cl^−^ as the newly interlayer anion. The characteristic diffraction peaks corresponding to the 003 and 012 planes can be well-indexed to Co_6_Al_2_CO_3_(OH)_16_·4H_2_O (JCPDS file No. 51-0045), which also reveals the partial substitution of Ca^2+^ by Co^2+^, and of CO_3_^2−^ by Cl^−^ in the reconstructive layered structures. 

However, such a reconstruction suggests that most of the Ca^2+^ or Al^3+^ ions in the non-neutral solution are precipitated as hydroxides, which still have specific LDH structures. Comparatively, the diffraction peaks of the Co-absorbed LDH at pH = 6 show extremely high background noises in the XRD pattern (Figure 1c), indicating the possible presence of an amorphous state of agglomerates. Generally, in a relatively neutral environment (pH = 6), Al^3+^ ions exist in the form of Al(OH)_3_. The relatively weakened diffraction peaks in Figure 1c can also be attributed to the agglomeration of amorphous phases. Having unique physical and chemical properties due to the disorder structures of amorphous materials, the investigation into such materials has become the forefront of condensed matter physics and is important in the design of new materials [16,17]. Amorphous Al(OH)_3_ is neither stable in strong acidic nor strong alkaline environments. For acidic environments, the similar adsorption mechanisms interacting between LDHs and divalent cations have been widely discussed in the literatures [11]. As for an environment with a pH of 12, Co (II) could precipitate in the form of Co(OH)_2_ prior to adsorption precipitation. In order to eliminate the interference of pH and to investigate the influence of CaAl-Cl-LDH on Co adsorption only, the pH was set to 6 in this research.

**Figure 2:**
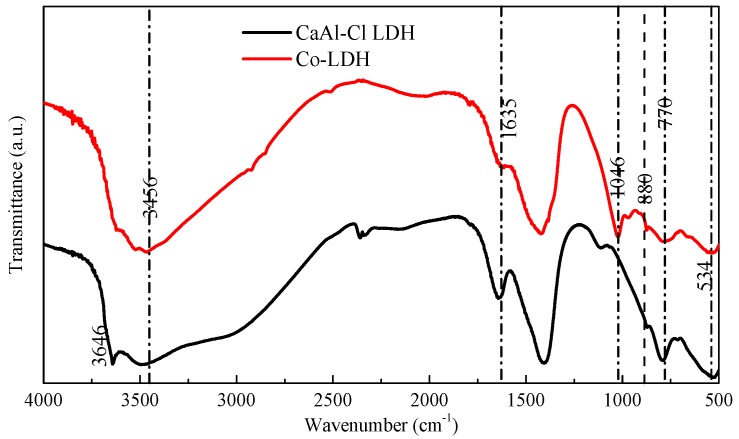
FT-IR spectra of CaAl-Cl-LDH and Co-adsorbed LDH at the concentration of 8 mM.

FT-IR measurements of CaAl-Cl-LDH and Co-absorbed LDH are carried out to clarify their component and structural characteristics, as shown in Figure 2. The peaks at 3646 cm^−1^ and 3456 cm^−1^ are related to the stretching vibrations of –OH groups in the lattice water and structural –OH groups in the CaAl-Cl-LDH [8]. The shoulder of the CaAl-Cl-LDH at 1420 cm^−1^ is attributed to CO_3_^2−^ by the incorporation of CO_2_ during the synthesis period [18]. Correspondingly, the newly emerged adsorption band at 1046 cm^−1^ is attributed to the carbonate anions of CO_3_^2−^ intercalated in the layered Co-LDH. The vibration around 1635 cm^−1^ can be assigned to the bending vibration of the HO–H that usually exists in interlayer water molecules. For Co-LDH, the band at around 1635 cm^−1^ is weakened, which indicates that fewer water molecules are inserted in the LDH lattice interlayer. This is consistent with the observation from the XRD analysis that the newly formed Co_6_Al_2_CO_3_(OH)_16_·4H_2_O has fewer water molecules in its stoichiometric formula. The peak at 880cm^−1^ is ascribed to the stretching vibration of [AlO_2_]^−^, which indicates that Al(OH)_3_ is stabilized in the initial CaAl-Cl-LDH or the final Co-adsorbed LDH under weak acidic conditions [19]. There are two wide absorption bands near the regions of 650−800 cm^−1^ and 3000−3750 cm^−1^, which are generally ascribed to the amorphous phases [20]. Either of the bands obtained at 770 cm^−1^ or 534 cm^−1^ are related to the stretching and bending vibration of Al-OH groups caused by Co^2+^ adsorption [18].

**Figure 3:**
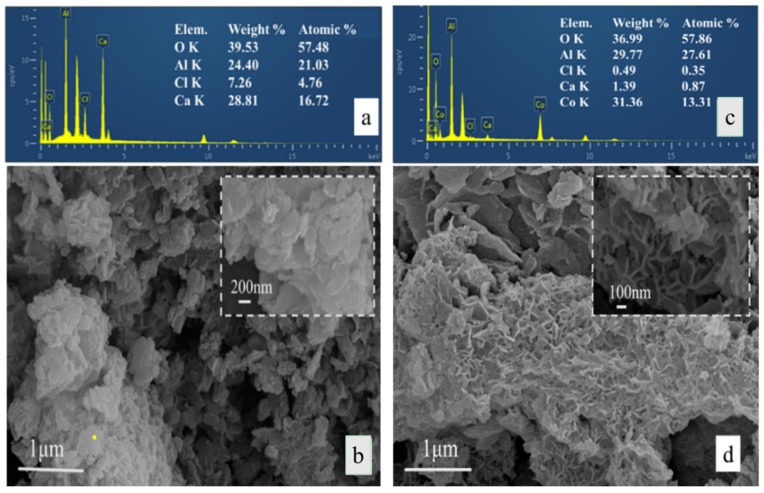
The energy dispersive X-ray spectroscopy (EDS) spectrum and SEM images of CaAl-Cl-LDH (**a**,**b**) and Co-LDH (**c**,**d**).

Figure 3 shows the SEM and EDS patterns of CaAl-Cl-LDH and Co(II)-LDH. The composition and quantity changes of O, Al, Cl, and Ca atoms in seaweed-like Co-LDH are compared by using an energy dispersive spectrometer (EDS). Accordingly, the initial Ca/Al molar ratio is found to be a bit lower, which is probably due to the co-existence of soluble Al(OH)_4_^−^ in the solution. The EDS also indicates a probable decrease in the content of the Ca and Cl elements for the CaAl-Cl-LDH after being Co(II)-adsorbed.

**Figure 4:**
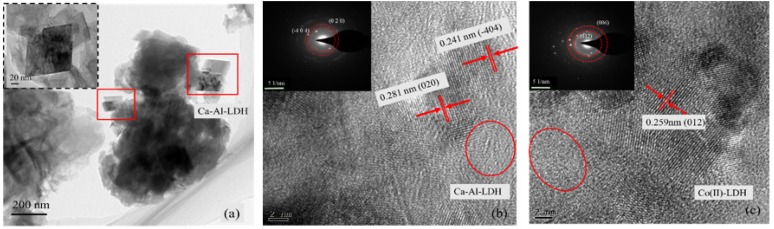
TEM image of CaAl-Cl-LDH (**a**) and HR-TEM images of Ca-Al-Cl-LDH (**b**) and Co-LDH (**c**).

In Figure 3c, there are several irregular hexagonal platelets stacked together, which have the dimensions of roughly 100–400 nm in diameter and 5–20 nm in thickness. The SEM results show that the morphology of the Co-adsorbed LDH is mostly an anomalistic lamellar structure with a coarse surface, that is poriferous and of a seaweed-like structure, and therefore it is different from the original LDH. Furthermore, the inter-lamellar spacing distance is sensitive to the structure variations between amorphous and crystalline LDH, which are also usually regarded as criteria for the cation adsorption capacity [21,22]. Consequently, the crystal structure evolution of CaAl-Cl-LDH before and after cobalt adsorption, including the phases, boundaries of the grains, and the domains, are investigated in Figure 4 using a high-resolution transmission electron microscope (HR-TEM).

As shown in Figure 4a, the original CaAl-Cl-LDH exhibits an irregular platelet morphology that is stacked in a random orientation. The presence of two-dimensional lattice fringes indicates that the CaAl-Cl-LDH has a kind of polycrystalline structure. Two typical inter-planar spacings, 0.281 nm and 0.241 nm, can be observed in Figure 4b. These two crystal parameters fit well with those of the (020) and the ($\bar{4}$04) planes of the Ca_4_Al_2_O_6_Cl_2_·10H_2_O, which further confirms the lattice information obtained from the XRD results (Figure 1). After Co adsorption, an obvious and new structural disorganization area is observed and the inter-planar distance is nearly 0.259 nm, corresponding to the (012) plane of the Co-LDH (Figure 1a). The newly found inter-planar distance may arise from the ionic size of cobalt being smaller than that of calcium, and this may be explained by Vergard’s law [23,24]. In addition, many amorphous phases coexisting with LDHs can be recognized in Figure 1b,c (red circle area), which is consistent with the observations from XRD and SEM-EDS analyses.

**Figure 5:**
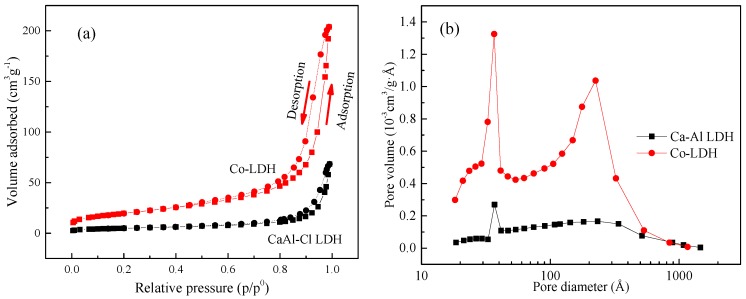
N_2_ adsorption and desorption isotherm loop (**a**) and Barrett-Joyner-Halenda (BJH) pore-size distribution (**b**) for CaAl-Cl-LDH and Co-adsorbed LDH (pH = 6).

The Brunauer-Emmett-Teller (BET) adsorption isotherm technique is applied to depict the porosity characteristics, including both the N_2_ adsorption/desorption isothermal process and the porosity parameters of the LDHs (Figure 5). The N_2_ adsorption-desorption isothermal of LDHs follows the type IV isotherm combined with the H_3_ type loop, which is also consistent with the characteristics of plate-like materials [25]. The values of the BET testing results of CaAl-Cl-LDH and Co-LDH are listed in Table 1. As shown in Figure 5 and Table 1, the pore size of CaAl-Cl-LDH particles is mainly distributed in the range of 3–4 nm (micropores), and the mean pore size is about 14 nm (mesopores). Generally, the micropores (<2 nm) and mesopores (2–50 nm) are the major factors in the adsorption of CaAl-Cl-LDH [26]. The presence of a small amount of macropores (>50 nm) within the original LDH, as shown in Figure 5, probably arises from the stacking of the plate-like CaAl-Cl-LDH within the inter-particle spaces. 

After Co adsorption (Table 1), the Co-adsorbed LDH exhibits an enlarged surface area (70.747 m^2^/g) and an increased total pore volume (0.317 cm^3^/g). The enlarged specific surface area indicates the modification of the surface structure properties of the Co-LDH, which is consistent with the SEM observation that the seaweed-like structure takes on mutually overlapping meshy plates or plate-like structures. Clearly, there is another factor besides the high surface area that leads to the improvement of the cobalt physical adsorption ability of the synthesized CaAl-Cl-LDH. 

**Figure 6:**
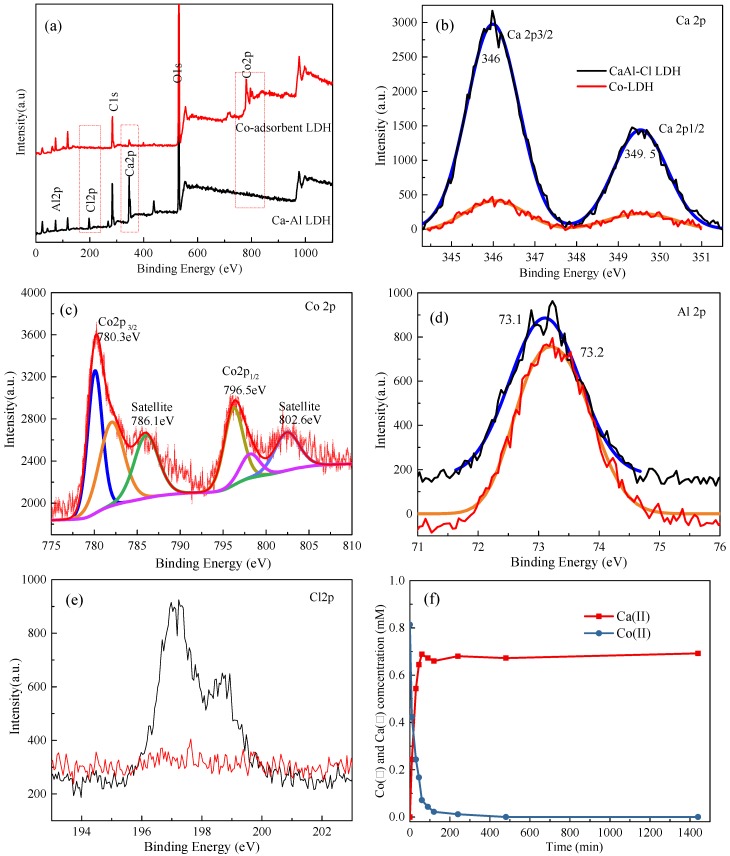
X-ray photoelectron spectroscopy (XPS) spectra survey of the CaAl-Cl-LDH before (black line) and after (red line) cobalt adsorption (**a**). The Ca 2p (**b**), Co 2p (**c**), Al 2p (**d**) and Cl 2p (**e**) spectra before and after cobalt adsorption. (**f**) The concentrations of free ionic Ca(II) and Co(II) in the solution during the removal of Co(II) with CaAl-Cl-LDH.

The XPS spectra of Ca-Al LDH before and after cobalt adsorption are shown in Figure 6. The C1s peak is attributed to correct all peaks and only the Ca, Co, Al, Cl, and O elements are presented. As shown in Figure 6b, the high-resolution spectrum of Co2p could be deconvoluted into two bonding states, splitting Co 2p_3/2_ and Co 2p_1/2_ with binding energies of 780.3 eV and 796.5 eV, respectively. Additionally, there are obvious shakeup satellite peaks for Co 2p_3/2_ and Co 2p_1/2_ observed at 786.1 and 802.6 eV, respectively, which confirms the existence of Co^2+^ [27,28]. The peaks of CaAl-Cl-LDH at 346 eV and 349.5 eV correspond to Ca 2p_3/2_ and Ca 2p_1/2_, respectively [29]. The binding energies of the Al 2p peak before and after cobalt adsorption were, respectively, 73.1 eV and 73.2 eV, indicating the same chemical environment. On the other hand, the inclusion of Co into the system was accompanied by a decrease in both Ca 2p and Cl 2p, demonstrating an interaction between Co and Ca in accordance with the EDS analysis. Furthermore, in accordance with the EDS results, Cl^−^ is no longer present in the Co-LDH, verifying that another type of anion—CO_3_^2−^—is incorporated into the newly formed LDH to balance the electric neutrality.

This interaction was further testified by measuring the concentrations of free ionic Ca(II) and Co(II) during Co(II) removal by CaAl-Cl-LDH, using inductively coupled plasma optical emission spectroscopy (ICP-MS). Co(II) decreases significantly from 0.814 to 0 mM as Ca(II) increases from 0 to 0.692 mM, as shown in Figure 6f. Assuming that Ca(II) is totally ion-exchanged by Co(II) in the CaAl-Cl-LDHs, the maximum exchange amount of Co(II) for CaAl-Cl-LDH is 0.692 mM/g. As observed in Figure 6f, there is an obvious difference between the total amount of Co(II) adsorption and Ca(II) desorption, which can be regarded as the Co(II) adsorption amount contributed by the existence of Al(OH)_3_. Moreover, it should be noticed that there is a certain loss of Ca(II) due to the slow formation of CaCO_3_ in the carbonization process. However, the ion-exchange process is the dominant reaction and, accordingly, it is accompanied by CaCO_3_ precipitation. Therefore, the loss content of the carbonization process should be smaller than that of the ion-exchange process, especially at the early stage. Taking the above observations into account, the adsorption mechanism during the Co(II) removal process is quite complex and can be ascribed to two aspects: the co-precipitation or surface precipitation process interaction between Co(II) and CaAl-Cl-LDH, and the surface complexation by amorphous Al(OH)_3_. 

**Figure 7:**
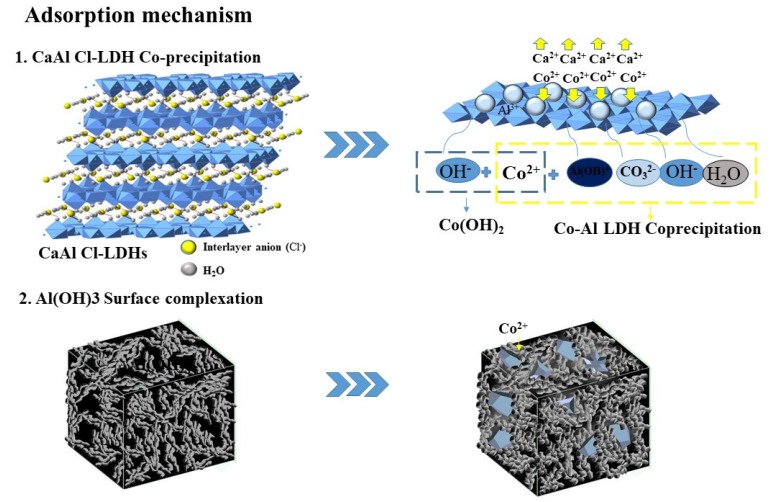
Scheme representing the Co(II) adsorption/absorption mechanisms of CaAl-Cl-LDH.

For the Ca-Al-Cl-LDH precipitation process, as discussed in the literature [30,31], the adsorption of divalent metal cations such as Co^2+^, Ni^2+^, Cu^2+^, or Zn^2+^ generally occurs through co-precipitation. To be specific, the CaAl-Cl-LDHs are firstly dissociated into charged particles (as shown in Equation (1)). Then, Co(II) could co-precipitate with the dissociative OH^−^, Al(OH)_4_^−^, and CO_3_^2−^ together in the form of Co_6_Al_2_CO_3_(OH)_16_·4H_2_O (see Equation (2)). Cations such as Ar^2+^, B^2+^, Mn^2+^, and Zn^2+^ have been removed by aluminum hydroxide due to surface precipitation in past studies [32,33,34,35]. A poorly crystalline and increased surface area of Co-LDH was found in XRD pattern and BET analyses, indicating that the Al(OH)_3_ surface hybridizes into an amorphous form at the surface of the LDH at pH = 6. As a result, parts of the Co^2+^ will be attached to the aluminum hydroxide by surface complexation. Furthermore, aluminum hydroxide could be stable at pH = 6 and the co-existing Al(OH)_3_ shows a strong adsorption capacity for Co^2+^. When the pH < 6, the chemical reaction of amorphous Al(OH)_3_ would be inhibited [36]. This mechanism could reasonably be confirmed by the microstructure characterization obtained from the above analysis. As for the surface complexation on Al(OH)_3_, the formation of ≡AlOCo^+^ is presented in Equation (3). According to the analysis above, the Co(II) adsorption/absorption mechanisms of CaAl-Cl-LDH is schematically depicted in Figure 7.

Ca_4_Al_2_Cl_2_(OH)_12_·4H_2_O ⇌ 4Ca^2+^ + 2Al(OH)_4_^−^ + 2Cl^−^ + 4(OH)^−^ + 4H_2_O (1)

6Co^2+^ + 2Al(OH)_4_^−^ + CO_3_^2−^ + 8OH^−^ + 4H_2_O ⇌ Co_6_Al_2_CO_3_(OH)_16_·4H_2_O (2)

≡AlOH^0^ + Co^2+^ ⇌ ≡AlOCo^+^ + H^+^ (3)

## 4. Conclusions

According to our comprehensive analysis, the formation of crystalline/amorphous blends corresponds to two adsorption mechanisms. The crystalline phases are identified as Co_6_Al_2_CO_3_(OH)_16_·4H_2_O, which is attributed to the co-precipitation process interaction between Co(II) and CaAl-Cl-LDH. The formation of amorphous phases is due to surface complexation on amorphous Al(OH)_3_ hydrolyzed from CaAl-Cl-LDH. This research indicates that CaAl-Cl-LDH is useful for the decontamination and immobilization of Co(II), and can be applied as a very promising and highly cost-effective adsorbent in the remediation of metal-contaminated soils as well as the decontamination of aqueous solutions. 

## Figures and Tables

**Table 1:** Cell parameters and Brunauer-Emmett-Teller (BET) analysis results of CaAl-Cl-LDH and Co-LDH.

Variables	Crystal Parameters				Surface Area (m^2^/g)	Pore Volume (cm^3^/g)	Average Pore Diameter (nm)
	a (nm)	c (nm)	Crystal Symmetry	Space Groups			
CaAl- LDH	0.985	1.690	Monoclinic	*P*2_1_/*c*	17.604	0.106	14.230
Co-LDH	0.308	2.280	Hexagonal	*R* $\bar{3}$ *m*	70.747	0.317	13.507
